# Chicago Medical Society

**Published:** 1874-07

**Authors:** Will T. Montgomery


***Chicago Medical Society.*** *Transactions at the Meeting of May* 18, 1874. Reported by WILL T. MONTGOMERY.

Regular semi-monthly meeting, May 18, in the parlor of the Gault House. President, Dr. Quine, in the chair. Special order, for the meeting: Lecture by Prof. De Lafontaine on the Spectroscopic Appearances of Blood.

The president referred to the continued absence of a portion of the committee on membership, and suggested that some action be taken in reference to it. Dr. Earle moved that a temporary committee be appointed, to act in the absence of the regular one. Carried. The president appointed as such committee, Drs. Paoli, Ingals and Strong.

The Society next listened to the lecture announced for the evening. In his lecture, the professor referred to the controversy between different investigators as to whether or not it is possible to distinguish by means of the spectroscope between fresh and old blood. He with Dr. Simon had instituted experiments for the purpose of determining this, and had used four specimens of blood as follows: First, cattle blood, two years old; Second, his own blood, four weeks old; Third, oxen blood, three days old; Fourth, blood two days old, from a man who died of hydrophobia. The fresh specimens present two dark bands in the spectrum—one in the green and one in the yellow. The old specimens present an additional band in the red.

This is very distinct in the specimen two years old, but in the specimen four weeks old it is merely a faint line. The three bands are invariably present in old blood, and the additional band in the red color of the spectrum is characteristic of it.

The professor referred to some peculiarities presented by the blood of the hydrophobia patient. It remained fluid longer than healthy blood, but decomposition began much earlier. Two days after the death of the patient, bacteria began to appear in the blood and multiplied very rapidly. Two varieties of the yeast plant or fungi were also discovered.

He thinks that the spectroscope is more reliable as a means of determining the character of blood than the microscope, and predicts for it a wide range of usefulness in the study of pathology. Says that it is a better test for bile in the urine than Pettenkoffer's. The lecturer, assisted by Dr. Simon, exhibited specimens of fresh and old blood, which members of the Society were invited to examine by means of the spectroscope after adjournment.

A vote of thanks was given the professor for his interesting lecture.

Dr. Worrell, President of the State Medical Society, having come in during the progress of the lecture, was introduced by Dr. Quine, and addressed the society in a happy complimentary strain.

After the disposal of miscellaneous business, a motion to adjourn prevailed.

